# Direct activation of oestrogen receptor- **α** by interleukin-6 in primary cultures of breast cancer epithelial cells

**DOI:** 10.1054/bjoc.1999.1097

**Published:** 2000-03-06

**Authors:** V Speirs, M J Kerin, D S Walton, C J Newton, S B Desai, S L Atkin

**Affiliations:** Departments of Medicine, Academic Surgical Unit, Medical Research Laboratory, University of Hull, Hull, HU6 7RX, UK; Scunthorpe General Hospital, Scunthorpe, DN15 7BH, UK

## Abstract

Interleukin 6 (IL-6) is secreted by breast tumours and shows synergistic activity with 17β-oestradiol (E2), leading to increases in reductive 17β-hydroxysteroid dehydrogenase activity in breast cancer epithelial cells. However, the mechanisms involved are poorly understood. Using short-term epithelial cultures established from primary breast tumours, we have examined whether IL-6 could directly affect transcriptional activity of oestrogen reception α (ERα). Tumour epithelial cultures were established from 15 breast tumours, grown to 70% confluence and transiently transfected with a plasmid reporter containing the vitellogenin oestrogen response element and the luciferase coding sequence (ERE-TK-LUC). Following transfection, cells were incubated with E2, IL-6, the pure anti-oestrogen ZM 182780 or combinations of these substances for 48 h. Luciferase activity was then measured in cell lysates. E2 caused a dose-dependent increase in luciferase expression, causing a maximum threefold stimulation at 100 p M. In the presence of IL-6, transcriptional activity was increased by up to 2.5-fold in ERα^+^cultures (11/15). In combination with E2, synergistic effects were observed with increases in luciferase activity of up to sixfold over controls. This effect could be blocked by treatment with ZM 182780. Pre-incubation of cells with an antibody directed against the signalling component of IL-6, gp130, was ineffective in blocking the E2 response. This antibody reduced, but did not completely block the effect of IL-6 either alone or in combination with E2, suggesting cross-talk between the two signalling pathways. In conclusion, these results provide evidence for direct transcriptional activation of ERα by IL-6. © 2000 Cancer Research Campaign


*British Journal of Cancer* (2000) **82**(7), 1312–1316
© 2000 Cancer Research Campaign
DOI: 10.1054/ bjoc.1999.1097, available online at http://www.idealibrary.com on IDEAL®

### Direct activation of oestrogen receptor-α by interleukin-6 in primary cultures of breast cancer epithelial cells

**V Speirs[1], MJ Kerin[2], DS Walton[1], CJ Newton[1], SB Desai[3] and SL Atkin[1]**

Departments of [1]Medicine and [2]Academic Surgical Unit, Medical Research Laboratory, University of Hull, Hull HU6 7RX, UK; [3]Scunthorpe General Hospital, Scunthorpe DN15 7BH, UK

**Summary** Interleukin 6 (IL-6) is secreted by breast tumours and shows synergistic activity with 17β-oestradiol (E2), leading to increases in reductive 17β-hydroxysteroid dehydrogenase activity in breast cancer epithelial cells. However, the mechanisms involved are poorly understood. Using short-term epithelial cultures established from primary breast tumours, we have examined whether IL-6 could directly affect transcriptional activity of oestrogen reception α (ERα). Tumour epithelial cultures were established from 15 breast tumours, grown to 70% confluence and transiently transfected with a plasmid reporter containing the vitellogenin oestrogen response element and the luciferase coding sequence (ERE-TK-LUC). Following transfection, cells were incubated with E2, IL-6, the pure anti-oestrogen ZM 182780 or combinations of these substances for 48 h. Luciferase activity was then measured in cell lysates. E2 caused a dose-dependent increase in luciferase expression, causing a maximum threefold stimulation at 100 pM. In the presence of IL-6, transcriptional activity was increased by up to 2.5-fold in $ER\alpha^+$ cultures (11/15). In combination with E2, synergistic effects were observed with increases in luciferase activity of up to sixfold over controls. This effect could be blocked by treatment with ZM 182780. Pre-incubation of cells with an antibody directed against the signalling component of IL-6, gp130, was ineffective in blocking the E2 response. This antibody reduced, but did not completely block the effect of IL-6 either alone or in combination with E2, suggesting cross-talk between the two signalling pathways. In conclusion, these results provide evidence for direct transcriptional activation of ERα by IL-6. © 2000 Cancer Research Campaign

**Keywords:** breast cancer; steroids; interleukin-6

The pleiotrophic inflammatory cytokine, interleukin 6 (IL-6) is emerging as a key factor involved in regulation of local oestrogen biosynthesis in breast tumours. It achieves this by activating three principal enzyme complexes involved in oestrogen production, namely, aromatase, steroid sulphatase and 17-HSD type I (Speirs et al, 1993; Reed and Purohit, 1997). Both the IL-6 gene and protein are present in breast tumours and breast primary cultures (Basolo et al, 1996; Green et al, 1997). Receptors for both IL-6 and its signalling component, gp130, have been detected both in breast tumours and breast cancer cell lines (Chiu et al, 1996; Crichton et al, 1996; Douglas et al, 1997). Although elevated serum levels of IL-6 do not appear to affect survival in breast cancer patients, a recent study of 60 patients has shown raised serum IL-6 in 27% of these patients compared to only 2% of age-matched controls (Asgeirsson et al, 1998).

Substantial clinical, epidemiological and laboratory data show that oestrogen is a potent mitogen in breast cancer. Oestrogen action is mediated by binding to its nuclear receptor, the ER. Once bound to ligand, ER dimerization occurs and the resulting homodimer then binds to oestrogen response elements (EREs) in the transcriptional control region of target genes, thus initiating transcription of oestrogen-responsive genes. Two ERs have been identified, the classic ER, now known as ERα (Green et al, 1986; Greene et al, 1986) and a second receptor, ERβ (Mosselman et al, 1996). ERβ is highly homologous to ERα, at the DNA (96%) and ligand binding (58%) domains, while the A/B domain, hinge region and F region are not well-conserved (Mosselman et al, 1996). Further work has indicated that in addition to homodimers, the α and β receptors can form DNA-binding heterodimers (Cowley et al, 1997; Pace et al, 1997).

As well as oestrogen-stimulated proliferation, the growth and metabolism of breast cancer cells is additionally modulated by growth factors/cytokines, which can be secreted under direct and indirect regulation of 17β-oestradiol (E2; Dickson et al, 1986). IL-6 is one such factor (Speirs et al, 1993; Duncan et al, 1994). There is evidence for cross-talk between ERα and growth factors with direct activation of ERα in response to IL-2 (Newton et al, 1994*a*) and IGF-I (Newton et al, 1994*b*; Lee et al, 1997) in ERα-positive breast and pituitary cell lines. Related to these observations, IL-6 has recently been shown to activate the androgen receptor in another hormone-sensitive cancer, prostate cancer (Hobisch et al, 1998).

Previously, we have shown that IL-6 strongly synergizes with E2, leading to increases in reductive 17β-hydroxysteroid dehydrogenase activity (and hence E2 production) in breast cancer epithelial cells (Speirs et al, 1993). A recent study showed a correlation in expression of IL-6 with ERα in breast tumours (Fontanini et al, 1999). It has also been reported that the inhibitory effects of IL-6 on breast tumour cells is dependent on ERα status: $ER\alpha^+$ cells

*Received 2 August 1999*
*Revised 5 November 1999*
*Accepted 8 November 1999*

*Correspondence to:* V Speirs, Molecular Medicine Unit, Clinical Sciences Building, St James's University Hospital, Leeds LS9 7TF, UK

respond to IL-6 while ERα⁻ cells were refractory to IL-6-mediated growth inhibition (Chiu et al, 1996). Based on these findings, we hypothesized that IL-6 may regulate activity of ERα in breast cancer. Therefore, the aim of the present study was to determine whether IL-6 could directly affect transcriptional activation of ERα using short-term primary cultures of human breast cancer epithelial cells as an in vitro model.

### METHODS

### Cell culture

Breast tumours (*n* = 15) were acquired directly from surgery and dispersed overnight in 0.1% collagenase type III (Life Technologies, Paisley, UK; Speirs et al, 1996*a*). All tumours were from post-menopausal patients and of ductal origin and grades 2 or 3. Eleven were positive for ERα. Ethical approval was obtained and all patients gave informed consent. Epithelial cells were isolated from the digest by differential centrifugation followed by culture in selective media (Speirs et al, 1998). These cells have previously been characterized by immunostaining, flow cytometry and genetic analysis and those derived from $ER\alpha^+$ tumours retain functional ER in vitro (Speirs et al, 1996*b*, 1998). Because of the reported weak oestrogenic effects of phenol red indicator found in standard tissue culture medium (Berthois et al, 1986), cultures were established in phenol red-free medium supplemented with 100 U $ml^{-1}$ penicillin, 100 μg $ml^{-1}$ streptomycin, 2 mM glutamine, 10 mM HEPES, 0.075% bovine serum albumin (BSA) (all Life Technologies), 10 ng $ml^{-1}$ cholera toxin (ICN Biomedicals, Oxon, UK), 0.5 μg $ml^{-1}$ hydrocortisone, 5 μg $ml^{-1}$ insulin and 5 ng $ml^{-1}$ epidermal growth factor (EGF) (all Sigma, Poole, UK). All subsequent experiments were conducted in this medium.

### Transient transfection

Freshly isolated epithelial cells were grown to 70% confluence (achieved after 3–5 days in vitro) in 6-well plates and transiently co-transfected using the cationic lipid, Lipofectamine (Life Technologies), with 5 μg of pSV β-galactosidase plasmid (Promega, Southampton, UK) and a plasmid reporter containing the vitellogenin ERE and the luciferase coding sequence (ERE-TK-LUC; Meyer et al, 1994) for 4 h at 37°C. Transfected cells were then incubated with either E2, IL-6 (10 ng $ml^{-1}$; Genzyme, West Malling, UK), the pure anti-oestrogen ZM 182780 (10 nM), anti-gp130 antibody (R&D Systems, Oxford, UK) or combinations of these substances. Appropriate positive (MCF-7 cells stably transfected with ERE-TK-LUC) and negative controls (primary cultures transiently transfected with a plasmid lacking the ERE) were set up in parallel. After 48 h, cultures were lysed and luciferase and β-galactosidase activities were measured. Luciferase activity was determined using a Report-a-Gene kit (Labtech International, Uckfield, UK) according to the manufacturer's instructions and quantified in a luminometer (Labtech International). β-galactosidase activity was measured by spectrophotometry at 420 nm. For each sample, luciferase activity was corrected for β-galactosidase activity and the final result expressed as percentage of untreated control. All experiments were repeated in triplicate.

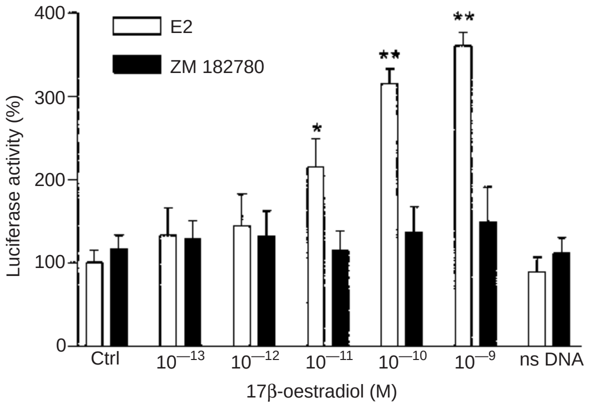

**Figure 1** Effect of increasing concentrations of 17β-oestradiol on luciferase activity in breast cancer epithelial cells and its antagonism by ZM 182780. ns DNA = plasmid lacking the ERE reporter. Each data point represents the mean + s.e.m. *$P < 0.05$, **$P < 0.002$ vs. control (Ctrl)

### ELISA

Levels of IL-6sR were quantitatively determined in conditioned medium (CM) by sandwich enzyme-linked immunosorbent assay (ELISA) (Boehringer Mannheim, East Lewes, UK). Breast cancer epithelial cultures from ten breast tumours were grown to 70% confluence, then incubated for 72 h with serum-free medium. CM was collected, centrifuged at 1000 rpm for 5 min to remove any particulate matter and stored at –80°C until analysed according to the manufacturer's instructions. The intra- and inter-assay variances were < 6% and < 12% respectively, with a sensitivity of 20 pg $ml^{-1}$ and the assay was not influenced by IL-6 up to 500 ng $ml^{-1}$. To correct for differences in growth rates of cultures established from different tumours, cultures from which the CM was collected were trypsinized and counted and the OD reading obtained for IL-6sR divided by cell number.

### Statistical analysis

All experiments were repeated in triplicate. Statistical analysis was performed using the Arcus software package for Windows (Research Solution, Cambridge, UK) on pooled data from individual cultures. The two-tailed Mann–Whitney *U*-test was used to test the difference between groups. Results were considered to be significant at $P \leq 0.05$.

### RESULTS

### Steroidal activation of ERα

Addition of E2 to $ER\alpha^+$ breast cancer epithelial cells transfected with an ERE linked to a luciferase reporter gene caused a dose-dependent increase in luciferase activity. A maximum threefold stimulation was observed at 100 pM (Figure 1). This is consistent with activation of ERα. To determine whether ERα was directly involved in increasing transcriptional activity, parallel cultures were incubated with the pure anti-oestrogen ZM 182780, which acts as an oestrogen antagonist. This completely abrogated the effects of E2 (Figure 1). No effects of E2 or anti-oestrogen were observed when similar cultures were transfected with a plasmid vector lacking the ERE, confirming the specificity of E2 for the response element.

© 2000 Cancer Research Campaign

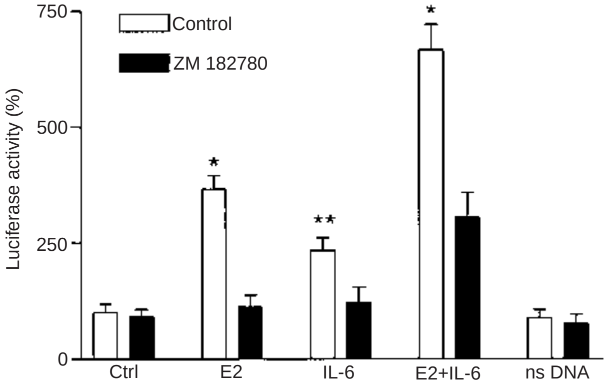

**Figure 2** Representative graph illustrating the mean effects + s.e.m. of E2 (100 pM) and IL-6 (10 ng $ml^{-1}$) alone or in combination and the antagonistic effects of ZM 182780 (10 nM) on luciferase activity in breast cancer epithelial cells. ns DNA = plasmid lacking the ERE reporter. $*P < 0.002$, $**P < 0.05$ vs. control (Ctrl)

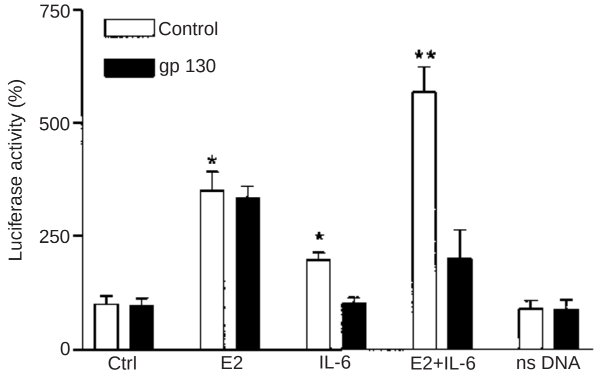

**Figure 3** Effects (mean + s.e.m.) of gp130 alone or in combination with E2 (100 pM) ± IL-6 (10 ng $ml^{-1}$) on luciferase activity in breast cancer epithelial cells. ns DNA = plasmid lacking the ERE reporter. $*P < 0.05$, $**P < 0.002$ vs. control (Ctrl)

### Activation of ERα by IL-6 and the effect of anti-gp130

Transcriptional activation of ERα was also observed in response to IL-6 (10 ng $ml^{-1}$), although the effect was much more modest, typically about two-thirds of the E2 response (Figure 2). No further increases in transcriptional activity in response to IL-6 were observed by increasing the concentration of this cytokine to a maximum of 25 ng $ml^{-1}$ (data not shown). As shown in Figure 2, when E2 (100 pM) and IL-6 (10 ng $ml^{-1}$) were added in combination, additive effects were observed with a sixfold increase in transcriptional activity. Addition of ZM 182780 significantly reduced the additive effects observed when E2 was combined with IL-6. Pre-incubation of cells with an antibody directed against gp130 was ineffective in blocking the E2 response and reduced but did not completely block the effect observed with IL-6 alone or in combination with E2 (Figure 3).

### Production of IL-6sR by breast cancer epithelial cells

A soluble form of the IL-6 receptor, IL-6sR, has been described which in contrast to other known cytokine soluble receptors, enhances the response of IL-6 in some biological systems (Mackiewicz et al, 1995). Therefore, IL-6sR was measured in CM samples collected from the epithelial cultures. IL-6sR was detected in all cultures, which produced a mean 41.12 pg $ml^{-1}$ IL-6sR (equivalent to 26.6 pg $ml^{-1}$ $10^{-6}$ cells; range 9.1–50.4). Although no statistically significant differences in production of IL-6sR were observed between $ER\alpha^+$ and $ER\alpha^-$ cultures, there was a suggestion of a trend for increased IL-6sR production by those which were $ER\alpha^+$.

### DISCUSSION

Using short-term primary cultures of breast cancer epithelial cells transiently transfected with the ERE-TK-LUC reporter plasmid as an in vitro model, this study has identified IL-6 as an activator of human ERα at the level of gene transcription. Further, although the signalling pathway of IL-6 is divergent from that of E2, this cytokine showed additive effects on transcriptional activity when combined with steroid. Our results complement and extend our previous work, which demonstrated additive/synergistic effects between IL-6 and E2 in regulating the reductive pathway of the steroid converting enzyme, 17-HSD type I (Speirs et al, 1993), and an association of IL-6 with other enzymes involved in modulating local oestrogen biosynthesis (Speirs et al, 1999).

As predicted, transcriptional activation of ERα was observed in response to E2. This was only observed in $ER\alpha^+$ cultures (11/15), indicating the requirement of ligand bound receptor to interact with the ERE. An increase in transcriptional activity from the same reporter was also observed in response to IL-6, although typically only about two-thirds of the levels observed with E2 were achieved. Again this was only evident in $ER\alpha^+$ cells. When IL-6 and E2 were co-incubated, additive/synergistic effects were observed. To determine if this was modulated via ERα, two experimental approaches were undertaken. First, the pure anti-oestrogen ZM 182370 was added as an antagonist to E2. Incubation with ZM 182370 blocked the effect of E2, either alone or when combined with IL-6. Unexpectedly, the anti-oestrogen also blocked the stimulatory effect of IL-6 added alone, suggesting that the effect of IL-6 may be independent of its receptor. To address this, the signalling component of the IL-6 receptor, gp130, was blocked using specific antibodies. The IL-6 receptor consists of an 80 kDa ligand-binding domain (gp80) and a 130 kDa signal-transducing protein (gp130) which lacks IL-6 binding activity. Association of ligand-bound gp80 with gp130 is necessary for signal transduction which is mediated by tyrosine kinases of the janus kinase family (Jaks) and STAT transcription factors (Kishimoto et al, 1992). When the IL-6 receptor was blocked with anti-gp130 antibodies the effect of IL-6 on transcriptional activity was inhibited; this had no effect on transcriptional activity of E2. The antibody also reduced, but did not completely block the cumulative effect of IL-6 and E2. Although the residual activity was less than that observed with E2 alone, this is probably mediated by E2 rather than IL-6 as blocking the gp130 signal transducer would prevent the formation of gp130 homodimers necessary for IL-6 signalling.

The presence of mRNA and protein for the complete IL-6 signalling system, i.e. peptide and gp80/gp130 receptors has been shown previously in human breast cancer biopsies (Crichton et al, 1996; Douglas et al, 1997; Green et al, 1997). A 55 kDa soluble form of gp80, known as IL-6sR, also exists. This is found in serum, is released into culture medium by the breast cancer cell line, MCF-7 and can be regulated by steroids and cytokines,

 © 2000 Cancer Research Campaign including IL-6 (Singh et al, 1995). By ELISA, we showed IL-6sR was also produced by our primary cultures. IL-6 can bind to IL-6sR augmenting its activity (Mackiewicz et al, 1995). The combination of these agents has been shown to enhance the cytostatic effects of IL-6 on T47D cells (Novick et al, 1992). By demonstrating the presence of IL-6sR in breast primary cultures, this may potentiate the interactive effects observed between IL-6 and E2.

When considered individually, E2 and IL-6 use different signalling pathways, which seem to be unrelated. While E2 binds to either ERα or ERβ, both members of the steroid receptor superfamily, with subsequent transcription via EREs, Jaks and STAT transcription factors mediate IL-6 signalling. However, the results of this study have shown there are clearly interactive effects. This complements a recent study in prostate cancer where another member of the steroid receptor superfamily, the androgen receptor, was also activated by IL-6 in reporter gene assays (Hobisch et al, 1998). Also in these assays, IL-6 showed synergistic activity with glucocorticoid via activation of the IL-6 response element on a rat α2-macroglobulin promoter (Takeda et al, 1998). This synergy was enhanced by the exogenous expression of glucocorticoid receptor, which parallels the results presented here; only cells which were $ER\alpha^+$ showed increased transcriptional activity in response to E2 and IL-6. An inhibition of protein kinase (PK) A, PKC and MAP kinase pathways also down-regulated IL-6-induced reporter gene activity (Hobisch et al, 1998), however, inhibitors of PKC failed to block growth factor-dependent activation, suggesting multiple pathways for ligand-independent activation of ERα (Ignar-Trowbridge et al, 1996). Other examples of ligand-independent activation of ERα include IL-2 and insulin-like growth factor (IGF)-I (Newton et al, 1994*a*, 1994*b*).

The role of IL-6 as a modulator of reductive 17β-hydroxysteroid dehydrogenase type I activity in breast tumours has previously been shown (Speirs et al, 1993). Since this enzyme is responsible for the production of E2, and as IL-6 can activate ERα, an established prognostic marker in breast cancer on which predictions to endocrine response and clinical outcome are based, our results suggest that cross-talk between these signalling pathways may have clinical significance. This may be particularly relevant in post-menopausal breast cancer patients where circulating levels of E2 are reduced. In those patients, ERα transcription may be activated by IL-6, which is known to be present in breast tumours (Speirs et al, 1996*b*; Reed and Purohit, 1997).

### ACKNOWLEDGEMENTS

We are grateful to Dr AE Wakeling of Zeneca, Macclesfield, UK for kindly supplying us with ZM 182780 and to Yorkshire Cancer Research and the Hull and East Riding Charitable Trust for supporting this work.

### REFERENCES

Asgeirsson KS, Olafsdottir K, Jonasson JG and Ogmunsdottir HM (1998) The effects of IL-6 on cell adhesion and E-cadherin expression in breast cancer. *Cytokine* **10**: 720–728

Basolo F, Fiore L, Fontanini G, Conaldi G, Calvo S, Falcone V and Toniolo A (1996) Expression and response to interleukin 6 (IL6) in human mammary tumours. *Cancer Res* **56**: 3118–3122

Berthois Y, Katzenellenbogen, JA and Katzenellenbogen BS (1986) Phenol red in tissue culture medium is a weak estrogen: implications concerning the study of estrogen-responsive cells in culture. *Proc Natl Acad Sci USA* **83**: 2496–2500

Chiu JJ, Sgagias MK and Cowan KH (1996) Interleukin 6 acts as a paracrine growth factor in human mammary carcinoma cells lines. *Clin Cancer Res* **2**: 215–221

Crichton MB, Nichols JE, Zhao Y, Bulun SE and Simpson ER (1996) Expression of transcripts of interleukin-6 and related cytokines by human breast tumors, breast cancer cells and adipose stromal cells. *Mol Cell Endocr* **118**: 215–220

Cowley SM, Hoare S, Mosselman S and Parker MG (1997) Estrogen receptors α and β form heterodimers on DNA. *J Biol Chem* **272**: 19858–19862

Dickson RB, McManaway ME and Lippman ME (1986) Estrogen-induced factors of breast cancer cells partially replace estrogen to promote tumour growth. *Science* **232**: 1540–1543

Douglas AM, Goss GA, Sutherland RL, Hilton DJ, Berndt MC, Nicola NA and Begley CG (1997) Expression and function of members of the cytokine receptor superfamily on breast cancer cells. *Oncogene* **14**: 661–669

Duncan LJ, Coldham NG and Reed MJ (1994) The interaction of cytokines in regulation of 17β-hydroxysteroid dehydrogenase activity in MCF-7 cells. *J Steroid Biochem Mol Biol* **49**: 63–68

Fontanini G, Campani D, Roncella M, Cecchetti D, Calvo S, Toniolo A and Basolo F (1999) Expression of interleukin 6 (IL-6) correlates with oestrogen receptor in human breast carcinoma. *Br J Cancer* **80**: 579–584

Green S, Walter P, Kumar V, Krust A, Bornert JM, Argos P and Chambon P (1986) Human oestrogen receptor cDNA: sequence, expression and homology to v-erb-A. *Nature* **320**: 134–139

Green AR, Green VL, White MC and Speirs V (1997) Expression of cytokine messenger RNA in normal and neoplastic human breast tissue: identification of interleukin-8 as a potential regulatory factor in breast tumours. *Int J Cancer* **72**: 937–941

Greene GL, Gilna P, Waterfield M, Baker A, Hort Y and Shine J (1986) Sequence and expression of human estrogen receptor complementary DNA. *Science* **231**: 1150–1154

Hobisch A, Eder IE, Putz T, Horninger W, Bartsch G, Klocker H and Culig Z (1998) Interleukin-6 regulates prostate-specific protein expression in prostate carcinoma cells by activation of the androgen receptor. *Cancer Res* **58**: 4640–4645

Ignar-Trowbridge DM, Pimentel M, Parker MG, McLachlan JA and Korach KS (1996) Peptide growth factor cross-talk with the estrogen receptor requires the A/B domain and occurs independently of protein kinase C or estradiol. *Endocrinology* **137**: 1735–1744

Kishimoto T, Akira S and Taga T (1992) Interleukin-6 and its receptor: a paradigm for cytokines. *Science* **258**: 593–597

Lee AV, C-N Weng, Jackson JG and Yee D (1997) Activation of estrogen-receptor mediated gene transcription by IGF-I in human breast cancer cells. *J Endocrinol* **152**: 39–47

Mackiewicz A, Wiznerowicz M, Roeb E, Karczewska A, Nowak J, Heinrich PC and Rose-John S (1995) Soluble interleukin 6 receptor is biologically active in vivo. *Cytokine* **7**: 142–149

Meyer T, Koop R, von Angerer E, Schonenberger H and Holler E (1994) A rapid luciferase transfection assay for transcription activation effects and stability control of estrogenic drugs in cell cultures. *J Cancer Res Clin Oncol* **120**: 359–364

Mosselman S, Polman J and Dijkema R (1996) ER-β: identification and characterisation of a novel human estrogen receptor. *FEBS Lett* **392**: 49–53

Newton CJ, Trapp T, Pagotto U, Renner U, Buric R and Stalla GK (1994*a*) The oestrogen receptor modulates growth of pituitary tumour cells in the absence of exogenous oestrogen. *J Molec Endocrinol* **12**: 303–312

Newton CJ, Buric R, Trapp T, Brockmeier S, Pagotto U and Stalla G (1994*b*) The unliganded estrogen receptor (ER) transduces growth factor signals. *J Steroid Biochem Mol Biol* **48**: 481–486

Novick D, Schulman LM, Chen L and Revel M (1992) Enhancement of IL-6 cytostatic effect on human breast carcinoma cells by IL-6 soluble receptor. *Cytokine* **4**: 6–11

Pace P, Taylor J Suntharalingam S, Coombes RC and Ali S (1997) Human estrogen receptor β binds DNA in a manner similar to and dimerises with estrogen receptor α. *J Biol Chem* **272**: 25832–25838

Reed MJ and Purohit A (1997) Breast cancer and the role of cytokines in regulating estrogen synthesis: an emerging hypothesis. *Endo Rev* **18**: 701–715

Singh A, Purohit A, Wang DY, Duncan LJ, Ghilchik MW and Reed MJ (1995) IL-6sR: release from MCF-7 breast cancer cells and role in regulating peripheral oestrogen synthesis. *J Endocrinol* **147**: R9–R12

Speirs V, Adams EF, Rafferty B and White MC (1993) Interactive effects of interleukin-6, 17β-estradiol and progesterone on growth and 17β-hydroxysteroid dehydrogenase activity in human breast carcinoma cells. *J Steroid Biochem Mol Biol* **46**: 11–15

© 2000 Cancer Research Campaign 

Speirs V, Green AR and White MC (1996*a*) Collagenase III: a superior enzyme for complete disaggregation and improved viability of normal and malignant human breast tissue. *In Vitro Cell Dev Biol* **32**: 72–74

Speirs V, Green AR and White MC (1996*b*) A comparative study of cytokine gene transcripts in normal and malignant human breast tissue and primary cell cultures derived from the same tissue samples. *Int J Cancer* **66**: 551–556

Speirs V, Green AR, Walton DS, Kerin MJ, Fox JN, Carleton PJ, Desai SB and Atkin SL (1998) Short-term primary culture of epithelial cells derived from human breast tumours. *Br J Cancer* **78**: 1421–1429

Speirs V, Walton DS, Hall M-C and Atkin SL (1999) In vivo and in vitro expression of steroid-converting enzymes in human breast tumours: associations with interleukin-6. *Br J Cancer* **81**: 690–695

Takada T, Kurachi H, Yamamoto T, Nishio Y, Nakatsuji Y, Morishge K, Miyake A and Murata Y (1998) Crosstalk between the interleukin-6 (IL-6)-JAK-STAT and the glucocorticoid-nuclear receptor pathway: synergistic activation of IL-6 response element by IL-6 and glucocorticoid. *J Endocrinol* **159**: 323–330

 *© 2000 Cancer Research Campaign*
